# Understanding Peripheral Bat Populations Using Maximum-Entropy Suitability Modeling

**DOI:** 10.1371/journal.pone.0152508

**Published:** 2016-12-09

**Authors:** Paul R. Barnhart, Erin H. Gillam

**Affiliations:** 1 Dickinson State University, Dickinson, North Dakota, United States of America; 2 North Dakota State University, Fargo, North Dakota, United States of America; University of Regina, CANADA

## Abstract

Individuals along the periphery of a species distribution regularly encounter more challenging environmental and climatic conditions than conspecifics near the center of the distribution. Due to these potential constraints, individuals in peripheral margins are expected to change their habitat and behavioral characteristics. Managers typically rely on species distribution maps when developing adequate management practices. However, these range maps are often too simplistic and do not provide adequate information as to what fine-scale biotic and abiotic factors are driving a species occurrence. In the last decade, habitat suitability modelling has become widely used as a substitute for simplistic distribution mapping which allows regional managers the ability to fine-tune management resources. The objectives of this study were to use maximum-entropy modeling to produce habitat suitability models for seven species that have a peripheral margin intersecting the state of North Dakota, according to current IUCN distributions, and determine the vegetative and climatic characteristics driving these models. Mistnetting resulted in the documentation of five species outside the IUCN distribution in North Dakota, indicating that current range maps for North Dakota, and potentially the northern Great Plains, are in need of update. Maximum-entropy modeling showed that temperature and not precipitation were the variables most important for model production. This fine-scale result highlights the importance of habitat suitability modelling as this information cannot be extracted from distribution maps. Our results provide baseline information needed for future research about how and why individuals residing in the peripheral margins of a species’ distribution may show marked differences in habitat use as a result of urban expansion, habitat loss, and climate change compared to more centralized populations.

## Introduction

Understanding the ecological and climatic factors that drive limitations of a species distribution is of fundamental importance for many conservation issues. Documentation of a species' distribution provides the baseline information needed for assessing range modifications, habitat use, genetic robustness, and conservation mitigation efforts [1–3]. A species distribution is also the fundamental characteristic used by local managers to employ species-specific environmental research, habitat management and biological reserve design [4].

Populations become increasingly fragmented or isolated at the periphery of a species' distribution [5] and individuals residing in these peripheral margins experience more challenging environmental conditions than their conspecifics in the center of the distribution [6]. Peripheral populations, especially those at or near the leading-edge of a distribution, are often more vulnerable to decline [7] and are of significant importance for conservation and management. Debate still exists as to the importance of peripheral populations in the evolution and persistence of a species [8]. Due to small population sizes, isolation, and the resulting threat of local extinction, some studies have concluded that these populations are unimportant for a species' persistence [9], while others argue that they contain important genetic information that natural selection can act upon [10].

Unfortunately, range maps are often too simplistic and lead to misinformed interpretation of a species' true distribution limits. These maps typically do not accurately depict the exact locations of peripheral populations, as most are simply polygons with no information about "islands" of species presence outside the continuous distribution [11]. Documenting exact occurrence locales of a species in the peripheral margins is critical for asking more advanced questions about ecology and behavior, as well as developing effective conservation plans [12] as they relate to peripheral population dynamics. This is particularly challenging when working with cryptic species that are often difficult to locate, resulting in unreliable information about species presence at different locations.

Insectivorous bats form a diverse group of mammals with complex ecological niches and habitat requirements. Although bats play key ecological roles in many ecosystems, conservation efforts can potentially be hampered due to lack of information on accurate occurrence information. Presence data for a given bat species is generally assessed through a combination of direct capture with mistnets and acoustic monitoring for echolocation calls, which can subsequently be identified at the species level. Although many studies have looked at the distribution and ecological requirements of individual bat species [13–17] only recently has statistical environmental modeling been incorporated into habitat studies [18–21]. Such modeling has the ability to accurately depict environmental data as they relate to species occurrence in a more streamlined and standardized way through GIS. Though these models do not accurately depict a species true distribution, as they do not include information such as biotic interactions, competition, human disturbances, and dispersal ability; they do depict suitable fine-scale habitat for that species, allowing regional managers to better target conservation efforts.

In North Dakota, eleven species of bats have been reported, of which three are listed as conservation priority by the state and one (*Myotis septentrionalis*) is federally listed as threatened. From a biogeographical viewpoint, North Dakota is an interesting location, as seven species (*Corynorhinus townsendii*, *M*. *thysanodes*, *M*. *ciliolabrum*, *M*. *septentrionalis*, *M*. *evotis*, *Lasiurus borealis*, and *M*. *volans*) reach the border of their IUCN distribution within the state [18]. Previous research on bats in North Dakota has been limited to studies reporting species occurrence in one area of the state and generally contain few capture records [19–26]. With the current threats facing bat populations, such a lack of region-specific information regarding species presence can have large management consequences. Due to North Dakota’s geographic position of straddling the line between the Midwest and the West, the state has a diversity of land types, many of which are also found in nearby states and provinces. Hence, studies assessing characteristics of peripheral populations in North Dakota can provide insight into the factors that commonly drive habitat selection in the northern latitudes of North America.

The major goals of this study were to: 1) document patterns of species presence through mistnetting throughout the state of North Dakota to determine if any of 7 bat species are found outside their IUCN distribution, and 2) identify key environmental variables driving region-specific species habitat suitability.

## Methods

### Ethics Statement

All procedures followed a protocol approved by the North Dakota State University Animal Care and Use Committee (Protocol # A12040). No animals were euthanized during this study and no federally protected species were sampled at the time of the study, as *Myotis sepetentrionalis* received its threatened status following the completion of this study.

### Data Collection

**Fig 1:**
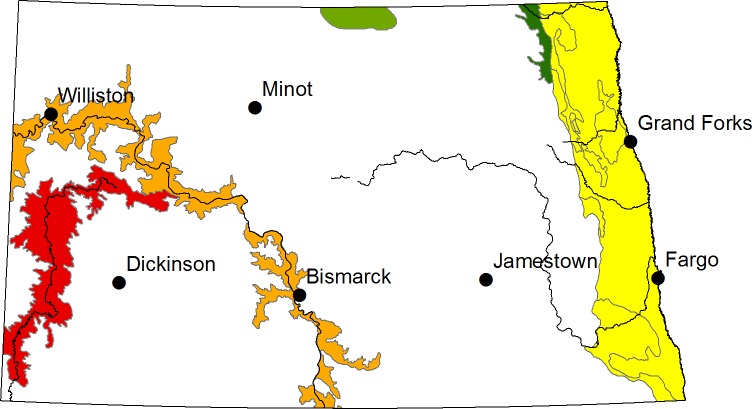
Map of North Dakota depicting the 5 sampling region in the state. Red = Badlands; Orange = Missouri River Valley; Light Green = Turtle Mountains; Dark Green = Pembina Gorge; Yellow = Red River Valley. Major cities are labeled for reference.

For sampling purposes, we divided North Dakota into 5 sampling regions: The Red River Valley, Pembina Gorge, Turtle Mountains, Missouri River Valley, and the Badlands of southwestern North Dakota (Fig 1). These regions spanned the entire state so that the boundary of each species distribution was sampled. The only regions of the state not sampled were the Drift Plains and Missouri Coteau, as previous sampling found that activity is particularly low in these regions and that there is a severe lack of natural roosting structures available (EHG and PRB, personal observations, 2009). A total of 17 locations were sampled across the 5 regions, with 4 to 7 nights of sampling within each location. At each location, we collected data at 3–7 sites, to ensure that we captured the diversity of habitats in the area. Selected sites spanned a variety of land types, including wildlife management areas, private land, state parks, federal parks, and wildlife refuges. At each site, we sampled using two methods: direct capture of bats via mist-netting and ultrasonic recording of echolocation calls from free-flying bats. Previous studies have found that using both mist-nets and ultrasonic detectors provides a more accurate estimate of species presence than either method alone [27].

A total of two to five mist-nets were deployed at each sampling site each night. At each site, the primary vegetation dominating the landscape (i.e. mixed ponderosa pine/juniper woodlands) was characterized. Mist nets were opened each night just before sunset and closed shortly before sunrise, or 2 hours after the last capture of a bat. Upon capture, we assessed the following standard measurements for each individual: species, sex, age, mass, forearm length, and reproductive condition.

Recordings of the echolocation calls of captured bats, which had been identified in the hand to species, were used to build a call library for analysis of unknown calls. To obtain these calls, captured bats were housed in clean cloth bags and transported to an open release site within 2 miles of the capture site. A 1.5" chemoluminescent tag (Rod-N-Bobb's Inc., Eau Claire, Wisconsin) was attached between the scapulae of the bat using non-toxic Elmer's glue. The release site was continually monitored for bat activity; when no bats had been detected for >60 seconds, one light-tagged individual was released and tracked with an ultrasonic bat detector (see Ultrasonic Detection below). All bats were released within two hours of capture.

Active ultrasonic detection was conducted on every sampling night using two broadband D240x Pettersson bat detectors (Pettersson Elektronik, Uppsala, Sweden). This time-expansion bat detection system records for a short period of time (1.7 to 3.4 seconds) and then broadcasts the recorded calls at one-tenth the original speed. Time-expanded calls were stored as an MP3 file on an iRiver player attached to the detector. Detectors were deployed as either passive or active systems. For the passive system, a D500x Pettersson bat detector was housed in a protective casing and placed within 2 miles of the netting site at a location containing high-quality bat habitat, such as a forest edge or riparian area. The protected bat detector was manually activated before sunset and automatically recorded sounds when an amplitude threshold was crossed. The active detection system involved monitoring and recording bat activity at mist-net sites using a second bat detector. All physical captures of specimens were done on private or protected land under the following permits: (1) The National Park Service (Permit Number: THRO-2009-SCI-0003); (2) North Dakota Game and Fish Department (Permit Number: GNF02778109); (3) North Dakota Parks and Recreation Department.

### Sound Analysis

Recorded echolocation calls were analyzed using Sonobat 3 ([28]; Sonobat, Arcata, CA). This system uses a decision engine based on the quantitative analysis of approximately 10,000 known recordings from species across North America to identify each recording to the species level. Since variation in call structure between geographic locations is a possibility, we also included our recordings from light tagging in the known recordings database. Sonobat 3 generates a spectrogram and measures 72 parameters that characterize call structure, such as highest frequency, lowest frequency, and duration of each individual call in the recorded sequence. We used only echolocation call sequences for species identification that had a 95% classification quality value or higher based on the algorithms employed in Sonobat 3 for analysis.

### Habitat Suitability Modeling

We used habitat suitability modeling or ecological niche modeling, to assess the habitat associations for ten bat species in the state (*Eptesicus fuscus*, *Lasionycteris noctivagans*, *Lasiurus borealis*, *L*. *cinereus*, *Myotis ciliolabrum*, *M*. *volans*, *M*. *septentrionalis*, *M*. *lucifugus*, *M*. *evotis*, and *Corynorhinus townsendii*). Although we documented 11 species in the state, low sample size for *M*. *thysanodes* (6 individuals) caused us to eliminate this species from further analysis. We used our presence data, gathered from both acoustic monitoring and physical capture, to delineate locations where each species is known to occur. Due to landscape heterogeneity seen across the study sites, we used high-resolution (30" or 1km^2^) raster datasets for all climatic modeling analysis.

We developed habitat suitability models using the program MaxEnt v.3.3.3 [29]. Such presence-only modeling has been shown to be very reliable and competitive with other high performing modeling techniques [30]. MaxEnt has also been shown to perform well with small samples sizes [31–32], which could prove useful for cryptic, volant species such as bats. This method produces probability density maps, where species presence is scored across small geographic areas as likely (score near 1) or unlikely (score near 0) [33].

For each species, we separated the presence data into test and training datasets (80% and 20%, respectively) and ran the jackknife validation function to minimize biases associated with small sample sizes. Because MaxEnt chooses which presence data to use in model training and testing, we ran 50 model replications and then averaged them into a single habitat suitability model for each species. Using the autofeatures function, we produced response curves and did a jackknife analysis to measure variable importance in each model. Eco-geographical data were selected from 19 "Bioclim" variables and other bioclimatic variables that describe monthly precipitation and temperature [34]. Selected variables were deemed ecologically relevant based on knowledge of the biology and annual activity patterns of North American bats [35]. The following variables were initially isolated for modeling: altitude; roads; rivers; annual precipitation (i.e. precipitation for each month); summer precipitation (i.e. precipitation for each month during summer); winter temperatures (i.e. temperatures for each month during winter); and a landcover dataset ([36]; http://www.mrlc.gov/nlcd06_ref.php; reclassified into 15 classes) that describes the "vegetational and artificial constructions covering the land surface" [37].

A fundamental assumption of MaxEnt is that the entire geographic area of interest has been sampled [38], yet this is typically not the case, as presence locations are more likely to be detected in areas that are more heavily surveyed. Because of this bias, background samples (i.e. pseudo-absence locations used by MaxEnt to determine areas of low suitability) used when developing habitat suitability models can have significant consequences on the model results [39]. For our study, a bias file was created allowing MaxEnt to only select background pseudo-absence locations within the same counties as the study took place. This provides MaxEnt with a pseudo-absence (i.e. bias file) file that has the same bias as the presence locations [40].

Models were run using the default MaxEnt settings, with the exception of the number of iterations (5000 instead of the default 500). To evaluate model complexity and reduce over-parameterization/over-fitting, we ran each model using different regularization betamultiplier values (1–12). These values affect the fitting of the output distribution, with large values being more generalized, geographically spread out; overall, this essentially acts as a smoothing parameter. This resulted in running 12 models for each species, (12 for each sampling technique). To find the most parsimonious models, we used AIC scores produced in ENMTools v. 1.3. To evaluate the correlation between these variables (i.e. test for multicollinearity), we used the variable correlation analysis in ENMTools v. 1.3 [41]. For variables that were highly correlated (R^2^ > 0.75), the less ecologically relevant variable was removed. Only variables that contributed more than 1% to the model were included in the final models. This resulted in eight final variables to be used for modeling: Altitude, roads, annual precipitation, rivers, mean October temperature, May precipitation, June precipitation, and landcover. The landcover dataset was reclassified into 15 classes (Table 1).

**Table 1:** Landcover reclassifications. Reclassifications from original landcover dataset were done based on current knowledge of the study sites and North Dakota ecoregion characteristics. These reclassifications are indicative of the North Dakota landscape and coincide with those officially used by the North Dakota Game and Fish Department.

Landcover Reclassifications
Mosaic cropland
Mosaic vegetation
Closed broadleaved evergreen forest
Closed broadleaved deciduous forest
Open broadleaved deciduous forest
Closed needleleaved evergreen forest
Open needleleaved deciduous or evergreen forest
Closed mixed broadleaved and needleleaved forest
Mosaic grassland
Closed shrubland
Closed herbaceous vegetation
Sparse vegetation
Broadleaved forest regularly flooded
Broadleaved forest or shrubland permanently flooded
Woody vegetation on regularly flooded or waterlogged soil

Using the statistical outputs of the most parsimonious MaxEnt models, we extracted the three variables for each species that had the most explanatory power in building the SDMs (Table 2). Using the final habitat suitability models for each species, we evaluated the amount of overlap between all 10 species using the niche overlap function in ENMTools v.1.3. We used the measure Schoener's *D* to evaluate the amount of overlap between species habitat suitability maps (Table 3). Schoener's *D* quantifies niche overlap from 0, meaning there is no overlap between habitat suitability maps (0% overlap), to 1, where all grid cells are of equal suitability for both species (100% overlap). We then qualitatively compared the models to look for areas throughout the state where modeling predicted areas of high suitability for multiple species. This analysis was conducted to provide information that could be used by managers to streamline conservation efforts and focus on managing areas that are important to the largest number of species.

**Table 2:** Three most relevant predictive environmental variables used for MaxEnt habitat suitability modeling for each species. Species abbreviations are as follows: *Corynorhinus townsendii* = Coto; *Lasiurus borealis* = Labo; *Myotis ciliolabrum* = Myci; *Myotis evotis* = Myev; *Myotis septentrionalis* = Myse; *Myotis volans* = Myvo. Landcover percentages represent the percentage amount for each 30arc second raster grid associated with each species. For example, Coto was associated, through MaxEnt modelling, to habitats that contained 50–70% mosaic grasslands, 20–50% forest or shrubland, were open (>15%) and contained sparse vegetation (<15%).

SPECIES	PREDICTIVE EGVS IN MAXENT MODEL
**COTO**	
**1**	Max Temperature of Warmest Month
**2**	Mosaic grassland (50–70%) / forest or shrubland (20–50%); Closed to open (>15%) (broadleaved or needleleaved, evergreen or deciduous) shrubland (<5m); Sparse (<15%) vegetation
**3**	Min Temperature of Coldest Month
**LABO**	
**1**	Max Temperature of Warmest Month
**2**	Mosaic grassland (50–70%) / forest or shrubland (20–50%); Closed to open (>15%) (broadleaved of needleleaved, evergreen or deciduous) shrubland (<5m); Sparse (<15%) vegetation
**3**	Annual Mean Temperature
**MYCI**	
**1**	Mosaic forest or shrubland (50–70%) / grassland (20–50%); Sparse (<15%) vegetation
**2**	Max Temperature of Warmest Month
**3**	Annual Mean Temperature
**MYEV**	
**1**	Mean Temperature of Coldest Quarter
**2**	Max Temperature of Warmest Month
**3**	Mosaic forest or shrubland (50–70%) / grassland (20–50%); Sparse (<15%) vegetation
**MYSE**	
**1**	Mosaic forest or shrubland (50–70%) / grassland (20–50%)
**2**	Max Temperature of Warmest Month
**3**	Mean Temperature of Warmest Quarter
**MYVO**	
**1**	Mosaic forest or shrubland (50–70%) / grassland (20–50%)
**2**	Annual Mean Temperature
**3**	Mean Temperature of Warmest Quarter

**Table 3:** Schoener's D niche overlap statistic for ten bat species in North Dakota (excludes M. *thysanodes*)

SPECIES	COTO	EPFU	LABO	LACI	LANO	MYCI	MYEV	MYLU	MYSE	MYVO
**COTO†**	1									
**EPFU**	0.688	1								
**LABO††**	0.804	0.794	1							
**LACI**	0.627	0.769	0.697	1						
**LANO**	0.695	0.928	0.831	0.784	1					
**MYCI†**	0.748	0.687	0.72	0.734	0.707	1				
**MYEV†**	0.738	0.548	0.661	0.554	0.564	0.784	1			
**MYLU**	0.666	0.719	0.690	0.87	0.734	0.776	0.624	1		
**MYSE†**	0.676	0.734	0.668	0.824	0.738	0.825	0.624	0.824	1	
**MYVO††**	0.714	0.777	0.720	0.793	0.770	0.869	0.685	0.809	0.869	1

†Species Documented outside known IUCN distribution

†† Species whose MaxEnt SDM depicts suitable habitat outside known IUCN distribution

## Results

### Species Habitat Associations

**Fig 2:**
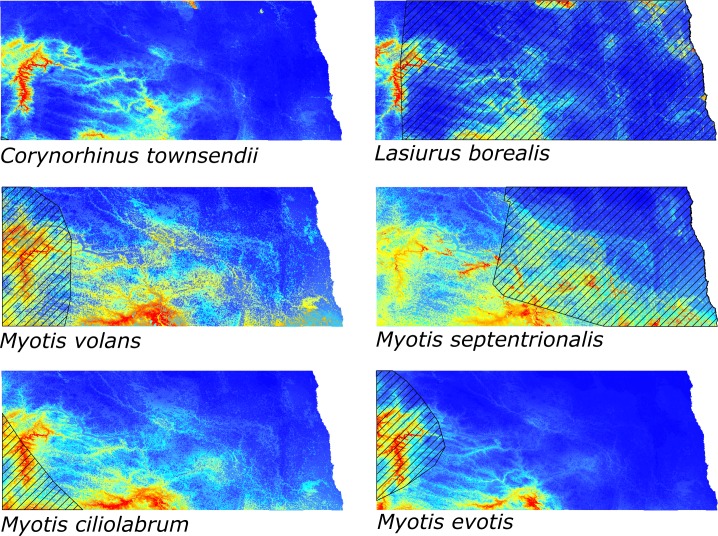
MaxEnt habitat suitability maps for six species found outside the known IUCN distribution in North Dakota. IUCN distribution depicted by black 10% hatch overlaid on habitat suitability map. Areas depicted as red are of high suitability and areas depicted as blue are of low suitability.

We documented eleven bat species in North Dakota, with seven having an IUCN distribution range limit intersecting the state (*C*. *townsendii*, *M*. *thysanodes*, *M*. *ciliolabrum*, *M*. *septentrionalis*, *M*. *evotis*, *L*. *borealis*, and *M*. *volans)*. Of these seven species, five were captured or recorded outside their known IUCN distribution (*C*. *townsendii*, *M*. *thysanodes*, *M*. *septentrionalis*, *M*. *ciliolabrum*, and *L*. *borealis*) and habitat suitability maps show areas of high suitability outside IUCN range limits of North Dakota for all six species analyzed (Fig 2).

Our capture/monitoring results confirm that *C*. *townsendii and M*. *thysanodes* are summer residents of North Dakota [25–26]. We positively identified *M*. *thysanodes* by three physical captures and three echolocation sequences, indicating this species is rare in the state. We also positively identified three *C*. *townsendii* by physical capture and >200 echolocation sequences from acoustic monitoring. *C*. *townsendii* was also acoustically detected in the Turtle Mountains region of North Central North Dakota, suggesting that this species may have gone undetected in more northern areas of the state or that its distribution is expanding to higher latitudes.

Statistical analysis within MaxEnt showed several important environmental variables for each species (Table 2). Maximum temperature of warmest month was one of the top three environmental variables used for habitat suitability model production for all but one species, *M*. *volans*. For all species, mosaic landscapes containing forests and shrubland were also important. However, *C*. *townsendii* and *L*. *borealis* were primarily associated with deciduous and needleleaf forests, while the myotids were more strongly associated with grasslands and shrubland open areas. Surprisingly, precipitation was not important for shaping the habitat suitability model in any species.

With the exception of *M*. *lucifugus*, Schroener's D statistic (Table 3) showed that *Myotis* species had the greatest habitat overlap with other *Myotis* species, with these four species associating with grasslands and/or shrublands. Interestingly, *M*. *lucifugus* had the greatest habitat overlap with *L*. *cinereus*. Of the remaining species that were either documented or predicted to be outside their known distribution in North Dakota, habitat suitability analysis showed that *C*. *townsendii* and *L*. *borealis* both were associated with deciduous and coniferous forest stands. Habitat suitability maps clearly showed four distinct areas in the state that contain highly suitable habitat for all species: 1) the badlands region of western North Dakota, 2) the Missouri River Valley in central North Dakota, 3) the Little Missouri Grasslands in western North Dakota, and 4) the Heart River in west-central North Dakota.

## Discussion

We positively identified five bat species outside their known IUCN distribution in North Dakota by either physical capture via mistnet or acoustic detection. This finding highlights the importance of continued monitoring efforts and the potential accuracy of many contemporary distribution maps, at least in the northern United States. Habitat suitability modeling revealed four key areas of the state that have significant roosting and foraging potential for all bat species. These include the Turtle Mountains in north-central North Dakota, the Missouri River (which bisects the state into eastern and western halves), the Heart River, and the Badlands region of western ND. The river systems that connect these areas could act as a migratory route between the species-rich badlands region and the comparably species-poor Missouri River Valley and Turtle Mountains regions. Given the ~2,400 mile length of the Missouri River, this river system may also allow for seasonal movement of bats from more centrally located populations into and out of peripheral areas. Finally, riparian corridors in these areas are dominated by aspen stands (*Populus spp*.)([42];PB, personal observation); given that the majority of North Dakota has limited tree cover, species might be using riparian corridors throughout the state to locate and exploit more diverse roosting and foraging resources [43–45]. These assumptions highlight the discernable differences between habitat suitability modelling and the development of a species distribution map. Information regarding biotic interactions, resource use, human modifications, and dispersal ability of a species are all needed to accurately depict a species true distribution. In the absence of such information, habitat suitability modelling allows researchers and managers to conduct region-specific research so that conservation decisions can be made in a timely fashion. Future research should be aimed at fulfilling the factors needed to develop accurate distribution maps as well as region-specific tasks such as monitoring the potential corridors to determine if bats are indeed using them as routes for movement between local foraging grounds and/or as migratory corridors for longer, seasonal movements.

Our study can serve as a baseline for comparing the habitat needs of bat species in northern regions of the United States and southern regions of Canada with such information from more centralized populations. Given the likely differences in landscape types and resource availability between populations separated by large distances, more regional information about a species’ needs are important for effective conservation. For example, *C*. *townsendii* has previously been reported to occur in riparian corridors, coniferous and deciduous forests, and avoid open grasslands [43–45]. This species is also known to preferentially roost in caves [43], although tree roosting has been documented [46]. Our study showed that this species was associated, through habitat suitability modeling, with grassland systems that are characteristic of the badlands region of North Dakota and our physical and acoustic captures documented this species in locations where caves are not known to occur, such as the Turtle Mountains and Missouri River Valley, suggesting that individuals are potentially exploiting different aspects of the habitat in the northern Great Plains. These findings suggest that *C*. *townsendii* has different foraging and roosting habitat preferences in the periphery of their distribution. However, other species, such as *M*. *ciliolabrum*, have previously been shown to occur in badlands terrain, juniper-pinyon stands, and coniferous and deciduous forests [47]. Our analysis seemed to confirm these finding in the peripheral margins of the species distribution. We found *M*. *ciliolabrum* and *M*. *septentrionalis* to be associated with grassland/shrublands (typical of the badlands terrain in North Dakota) and tall hardwood forests, respectively. These findings are similar to habitat preferences of this species in more central populations, suggesting that *M*. *ciliolabrum* and *M*. *septentrionalis* are not altering their habitat preferences along the peripheral margins [47–51]. This is of particular importance due to the April 2015 listing of *M*. *septentrionalis* as federally threatened [52], and little information is known about the habitat needs of this species throughout much of its range. Further fine-scale habitat analysis, especially information gathered on roosting preferences, diet, and micro-habitat, for bats, is needed for a thorough quantitative comparison between central and peripheral populations. Further work needs to be done to confirm such differences and the potential for exploitation of man-made structure as roosting resources.

The identification of five species outside their known IUCN distributionhighlights the importance of continuous monitoring of population trends and distributions, especially when relying on range maps constructed from simple polygons, which do not reflect changing characteristics of populations from the interior towards the periphery. Two species (*M*. *thysanodes* and *C*. *townsendii*) were documented far outside their IUCN distributions, which did not originally include North Dakota as part of their range. This documentation of species outside of their known ranges could represent two scenarios. First, these species have previously occupied these areas and simply went undetected during the limited monitoring studies that have been conducted in the past. Second, these species have expanded their distribution range limits since the time of the last monitoring study. Our results cannot distinguish between these two scenarios, but instead highlight that much information about species occurrence is still incomplete for many bats, and habitat suitability modeling coupled with monitoring surveys can greatly influence the contemporary knowledge of a species’ range limits. With imminent threats, such as white-nose syndrome and wind energy development, such oversight could have significant impacts on bat populations, especially those populations along the periphery of the species distribution. The use of habitat suitability modeling can allow managers and researchers to better identify areas that contain a higher likelihood of species presence, creating more targeted and effective management plans. Though habitat suitability does not depict the actual distribution of a species, it provides much more detailed region-specific information on the biotic and abiotic factors that are driving species occurrence. This will allow managers and researchers to not only ask more advanced questions about species in a particular region but will also allow for the comparison between peripheral and central populations. The latter can provide great insight as to the plasticity of a species which is of great importance as habitat continue to change.

Using the described modeling methods, we documented the top three environmental variables driving distributions for each species in North Dakota. Though there were discrepancies as to which variables were important for each species’ model, the importance of mixed heterogeneous habitat was common for all species. Temperature was an especially important climactic factor driving habitat suitability models, although, interestingly, precipitation was not. These findings suggest that temperature shifts associated with global climate change will likely be a critical factor in determining how the contemporary distributions of bat species will change over time.

An additional problem for contemporary modeling studies is that in most cases it is difficult, if not impossible, to quantitatively compare results of past studies that have primarily made qualitative assessments with those studies using data from GIS raster datasets and habitat suitability analyses. The majority of literature we examined for background information defined habitat associations loosely, typically only describing the immediate vegetation with little or no insight into the surrounding forest stand. Due to these discrepancies, it is difficult to make highly reliable comparisons between modern and historical environmental analysis. Habitat suitability modeling is becoming widely used in many taxa, including bats [53–54, 35]. Recently, other environmental suitability models have been produced, as well as ensemble modelling [55] which have the potential to more accurately depict habitat associations. As habitat suitability modeling continues as an important ecological tool, it is imperative that researchers obtain information that can be used collaboratively by others to provide a baseline for habitat use comparisons. Such information may be critical in the face of global climate change and increasing concerns about effective conservation of bat species, as well as other taxa.
